# Acute hepatitis C virus (HCV) infection in the setting of HIV coinfection: a single-centre 10-year follow-up

**DOI:** 10.7448/IAS.17.4.19638

**Published:** 2014-11-02

**Authors:** Patrick Ingiliz, Katharina Steininger, Marcel Schuetze, Stephan Dupke, Andreas Carganico, Ivanka Krznaric, Andreas Wienbreyer, Axel Baumgarten

**Affiliations:** Infectiology, Medical Center for Infectious Diseases, Berlin, Germany

## Abstract

**Introduction:**

Acute hepatitis C virus (HCV) infection has emerged as a major cause of morbidity in the HIV-infected population. Most guidelines recommend early treatment with pegylated interferon and ribavirin due to higher cure rates. The impact of acute HCV and its treatment on the outcome of the HIV-infected population is unknown. With new treatment options for HCV, early treatment of acute HCV has to be questioned. Here, we report a long-term analysis on patients with acute HCV.

**Methods:**

Retrospective analysis from an outpatient clinic from Berlin. All patients with the diagnosis of acute HCV according to The European AIDS Treatment Network (NEAT) criteria were included in the database at their date of HCV diagnosis and followed-up until the last medical contact. Demographic data was taken from the medical file. A fibrosis estimation was performed using transient elastography (Fibroscan(®). A Fibroscan value above 7 kPa was considered as significant fibrosis, above 12.4 kPa as liver cirrhosis. The following outcomes were documented: (a) liver-related: hepatic decompensation, cirrhosis, hepatocellular carcinoma. (b) non-liver-related: death, myocardial infarction, AIDS-defining event, psychiatric hospitalisation.

**Results:**

A total of 207 cases of acute HCV infection in HIV-infected patients were diagnosed between May 2002 and September 2013. All patients were male and declared homosexual contacts as their risk factor for having acquired HIV. The mean age was 39 years, 162 patients (78%) were on antiretroviral treatment, and the median CD4 cell count was 593/mm^3^ (IQR 443–749). At HCV diagnosis, the highest median alanine aminotransferase (ALT) level was 408 IU/mL (159–871), the HCV viral load was 800,000 IU/mL (45x103–2.8x106). 22 cases (11%) cleared their infection spontaneously, 161 (77%) underwent interferon-based treatment. Of those treated, 58% had a sustained virological response. 36 cases of HCV reinfection were documented. All patients were followed-up over an interval of 806 patient-years. The median liver stiffness was 5.3 kPa (4–7) after a median interval of 34 months. 33 patients developed significant fibrosis, and 11 patients (6%) developed cirrhosis. Nine (5%) patients died during follow-up, and 11 developed non-liver-related endpoints. All but one deceased patients had interferon-based treatment.

**Conclusions:**

Severe hepatic disease and death rarely occurs in a highly treated HCV population. As interferon-based treatment may induce side effects, it is from now on justified to await new HCV treatment options.

